# Storage Potential of Soil Functional Carbon Fractions in the World's Largest Plantations

**DOI:** 10.1002/advs.202504995

**Published:** 2025-08-07

**Authors:** Siyu Ren, Hui Wang, Tao Zhou, Chuankuan Wang, Zhenghu Zhou, Shirong Liu

**Affiliations:** ^1^ Institute of Carbon Neutrality Key Laboratory of Sustainable Forest Ecosystem Management‐Ministry of Education School of Ecology Northeast Forestry University Harbin 150040 China; ^2^ Heilongiang Maoershan Forest Ecosystem National Observation and Research Station School of Ecology Northeast Forestry University Harbin 150040 China; ^3^ Ecology and Nature Conservation Institute Chinese Academy of Forestry Key Laboratory of Forest Ecology and Environment of National Forestry and Grassland Administration No. 2 Dongxiaofu, Haidian District Beijing 100091 China

**Keywords:** carbon storage, forest management, mineral‐associated organic carbon, plantation, soil organic carbon

## Abstract

The expansion of planted forests is one of the most effective natural climate solutions. However, increasing forest age and climate change lead to weaken biomass carbon sink, highlighting the urgency of improving the storage of functional soil organic carbon (SOC) to maintain a high level of carbon sink on a long‐time scale. Using data from 501 planted forests and 564 natural forests across China, it is found that climate warming and drying increase the loss of topsoil (0–20 cm) mineral‐associated (MAOC) and particulate organic carbon (POC) during the conversion of natural forests to plantations. High‐precision random forest models for MAOC (77%) and POC (75%) derive the storage of the functional SOC in planted and natural forests. The total deficit of SOC in plantations compared to natural forests is $2.17_{1.87}^{2.52}$ Pg SOC (mean with 5% and 95% quantile). This substantial potential can be gained by plantation management, such as close‐to‐nature management. The minimal (14.61 Pg C), intermediate (22.36 Pg C), and ambitious (29.79 Pg C) objectives of SOC storage potential, providing science‐based targets for enhancing soil carbon sinks, are also estimated. Overall, these findings provide a transferable framework to assess functional SOC benchmarks and targets for policymakers and highlight the strategic role of plantation soils for global carbon neutrality goals.

## Graphical abstract

Climate warming and drying increase the loss of mineral‐associated and particulate organic carbon during the conversion of natural forests to plantations. Based on a survey of the world's largest plantations, the study quantifies the soil carbon deficit in plantations and assesses benchmarks and targets for functional soil organic carbon, highlighting the strategic role of plantation soils for global carbon neutrality goals.

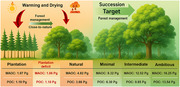

## 1 Introduction

Reforestation and afforestation with fast‐growing and stress‐resistant species contribute to the rapid increase in forest cover.^[^ ^1^ ^]^ Consequently, the expansion of planted forests is one of the most effective natural climate solutions.^[^ ^2^ ^]^ For example, forestation could contribute about 80% of the forest biomass carbon (C) sink in China since the 1980s.^[^ ^3^ ^]^ The global forest carbon stock was estimated at 662 Pg C; however, around 45% of this was stored in soils.^[^ ^4^ ^]^ Although there is widespread agreement on the need to understand how forest management changes soil organic carbon (SOC) storage, uncertainties and debates persist regarding the SOC dynamics under these “artificial” forestry projects.^[^ ^5^, ^6^, ^7^ ^]^ Especially, on a long‐term scale, SOC sequestration potential may play a more important role in mitigating climate change because the current forest age structure will decrease the biomass carbon sinks soon.^[^ ^8^ ^]^

Soil particulate organic carbon (POC), with a short turnover time, comprises structurally complex plant residues protected by biochemical recalcitrance and aggregates, while mineral‐associated organic carbon (MAOC), with a long turnover time, consists of low molecular weight organic compounds that are bound to soil mineral surfaces.^[^ ^9^, ^10^, ^11^, ^12^, ^13^ ^]^ Separating SOC into MAOC and POC provides a useful framework for predicting the response of SOC to environmental change.^[^ ^14^, ^15^ ^]^ Several previous meta‐analyses have quantified the response of bulk SOC to land use change or forest conversion.^[^ ^16^, ^17^, ^18^ ^]^ However, changes in soil MAOC and POC during the conversion from natural forests to plantations in different regions, as well as their covariations with climate and edaphic factors, have not been well quantified. This lack of understanding hinders our ability to predict SOC dynamics under forest management and climate change, particularly given the contrasting turnover times of MAOC and POC.

Additionally, estimating the potential for additional carbon storage (the difference between current and potential carbon) and its spatial distribution is crucial for guiding on‐the‐ground decision‐making regarding how and where to implement climate change mitigation strategies.^[^ ^19^ ^]^ Quantile multiple regression or random forest methods, which consider multiple factors that may constrain SOC storage, can be used to estimate the distribution of carbon storage potential based on the assumption that soils with similar external properties and identical long‐term management should support similar SOC levels.^[^ ^20^, ^21^ ^]^ Such methods can help quantify whether the capacities of MAOC and POC in plantations could reach the levels of natural forests. Addressing these knowledge gaps is essential for gaining a comprehensive understanding of how plantation soils function as carbon sinks in the context of global climate change, and for guiding plantation management practices aimed at enhancing SOC storage capacity.

**Figure 1:**
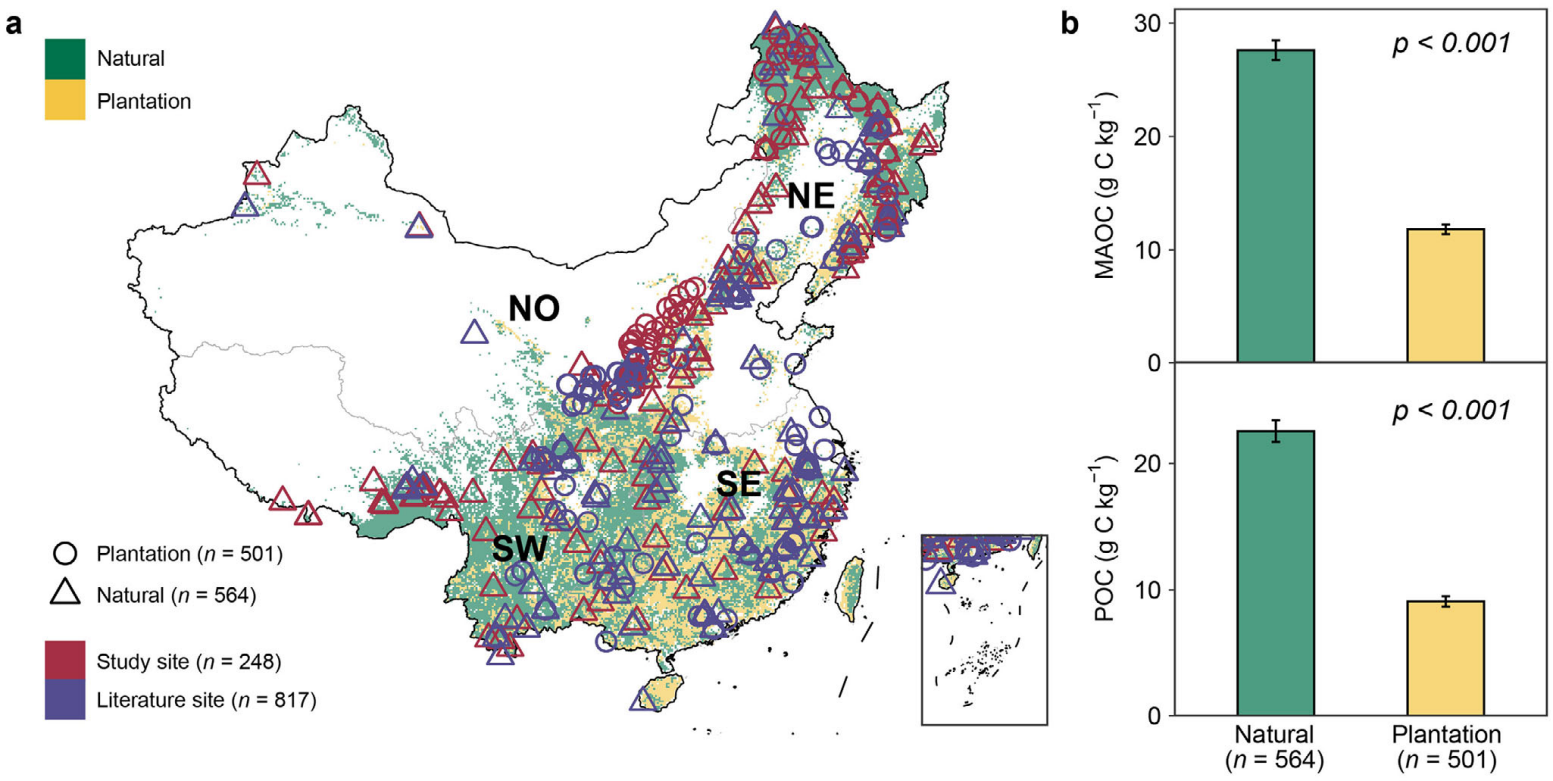
Site distribution and comparison of soil functional carbon fractions in planted and natural forests. a) Sample points from our national‐scale survey (*n* = 248) and literature synthesis (*n* = 817). SE, Southeast China; SW, Southwest China; NO, Northern China; NE, Northeast China. Detailed site information is provided in the Supplementary Data. b) The one‐way analysis of variance shows the differences in mineral‐associated organic carbon (MAOC) and particulate organic carbon (POC) between natural and planted forests (mean ± SE). The *p*‐value considered significant when *p* < 0.05.

China has the world's largest area of planted forests, ≈80 million hectares, which represents more than one‐quarter of the global planted forest area.^[^ ^22^, ^23^ ^]^ Although China's young forests currently act as a robust biomass carbon sink due to widespread afforestation and reforestation efforts, ongoing forest aging and climate change inevitably weaken this sink capacity in the future.^[^ ^24^, ^25^, ^26^ ^]^ Previous studies suggest that forests continue to accumulate carbon in soil during stand development despite the decline of biomass carbon sink, even in old‐growth stages.^[^ ^27^ ^]^ Therefore, SOC sequestration is critical to maintaining the high carbon sink of China's forests over the long term. To gain insight into this, a national‐scale survey in China (248 cases) and a literature synthesis (817 cases) were conducted to quantify the distributions of topsoil (0–20 cm) MAOC and POC in China's natural and planted forests (**Figure** **1**a). Our national‐scale survey included 165 and 83 cases for natural and planted forests, respectively, while the literature synthesis included 399 and 418 cases for natural and planted forests, respectively (Figure 1a). We aimed to estimate the spatial distributions and potential mechanisms of soil MAOC and POC in both natural and planted forests, explore the deficits of soil MAOC and POC in the world's largest plantation compared to natural forests, and quantify the storage potential of soil MAOC and POC in China's forests. Given China's leading role in global afforestation efforts, understanding MAOC and POC dynamics in this region is crucial for developing forest management strategies that are applicable worldwide.

## 2 Results and Discussion

### 2.1 Climate Warming and Drying Increase the Magnitude of Soil Carbon Loss During the Conversion of Natural Forests to Plantations

**Figure 2:**
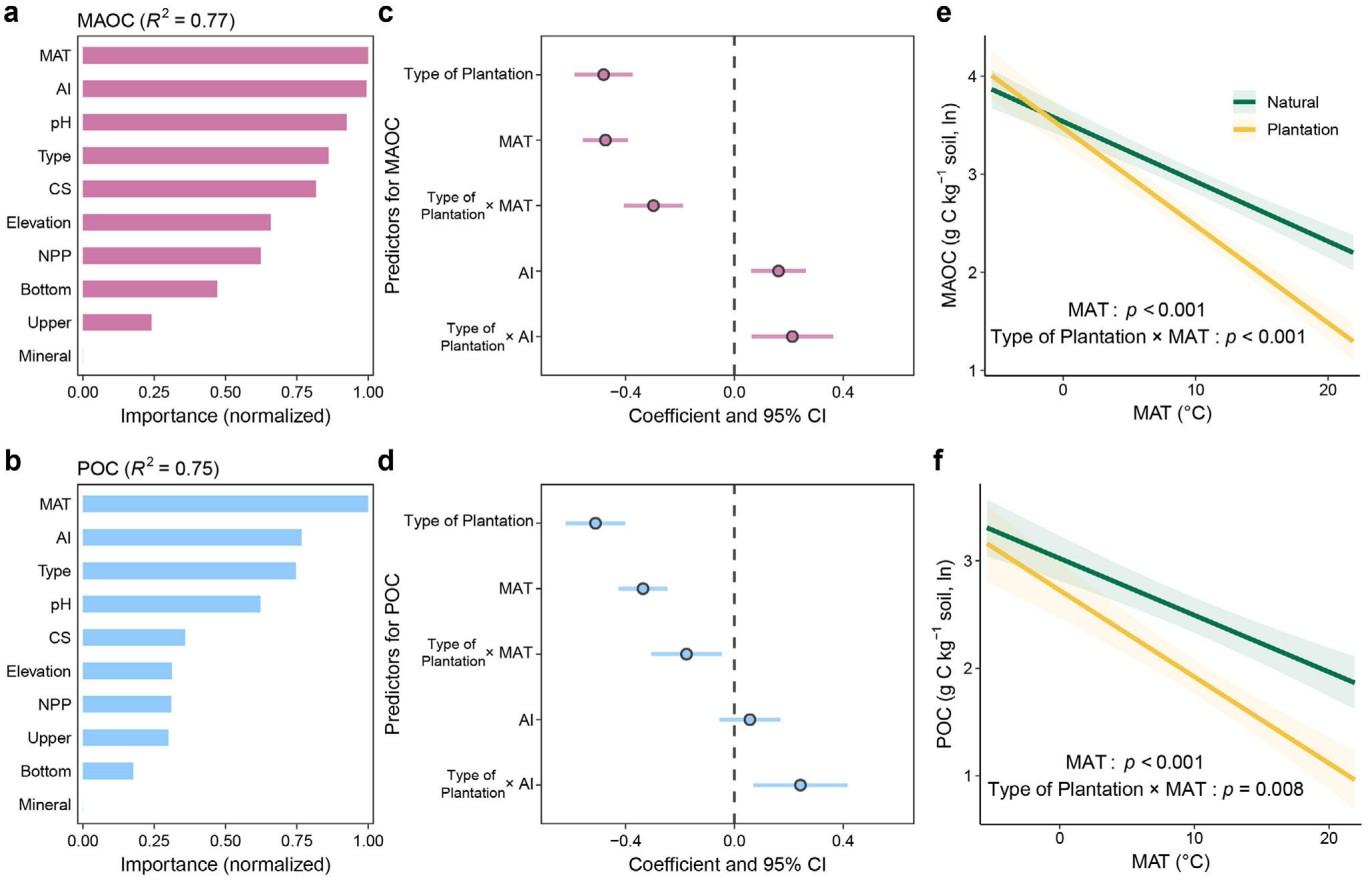
Contrasting climate responses of soil functional carbon fractions in planted versus natural forests. a,b) Normalized importance of predictors for MAOC and POC, respectively. MAT, mean annual temperature; AI, aridity index; CS, clay plus silt content; NPP, net primary productivity; Type, forest type (planted and natural forests); Upper, the upper depth of soil layer; Bottom, the bottom depth of soil layer; Mineral, high‐ and low‐activity type minerals. c,d) The linear mixed‐effects models (see Experimental Section) for MAOC and POC displaying coefficients (dots) and 95% confidence intervals (CI, error bars) for climate (MAT and AI) and interaction effects with forest type (plantation vs natural forest). The fixed and random effects collectively explained 79% and 74% of the total variance for MAOC and POC, respectively (*n* = 1065 for MAOC and POC observations). Both functional carbon fractions were natural‐log‐transformed, and all predictors were standardized. The negative coefficient for type of plantation (relative to natural forest) indicates that functional carbon content in plantations was significantly lower than in natural forests (*p* < 0.001). In addition, a negative correlation was observed between MAT and carbon contents (MAOC, *p* < 0.001; POC, *p* < 0.001), and a positive correlation was observed between AI and carbon contents (MAOC, *p* < 0.001; POC, *p* = 0.320). The significant interactions for type of plantation with MAT (MAOC, *p* < 0.001; POC, *p* = 0.008) and AI (MAOC, *p* = 0.005; POC, *p* = 0.006) showed that increasing MAT and decreasing AI had a greater negative impact on carbon contents in plantations than those in natural forests. e,f) Marginal effects of MAT on MAOC and POC from mixed‐effects models, showing stronger negative responses in plantations than in natural forests. Shaded bands represent 95% confidence intervals. The *p*‐values reflect the outcome of the optimal multivariate mixed‐effects model, with effects considered significant when *p* < 0.05.

One‐way analysis of variance showed that MAOC and POC concentrations in natural forests were 2.3 and 2.5 times higher than those in plantations (Figure 1b), respectively. In addition, the coefficients for type of plantation were negative in the mixed effect models, further suggesting that the plantation had significantly lower MAOC or POC than natural forests (**Figure** **2**c,d). The lower SOC in plantations is traditionally attributed to lower carbon inputs (lower net primary productivity, litterfall, and fine root biomass) and higher carbon outputs induced by a warmer soil microclimate under plantations.^[^ ^28^, ^29^, ^30^ ^]^ Compared with natural forests, plantations have fewer reactive iron (hydr)oxides^[^ ^31^, ^32^ ^]^ and cations,^[^ ^31^, ^33^, ^34^ ^]^ which would decline the formation of organic‐mineral and organic‐cation‐organic associations.^[^ ^35^, ^36^, ^37^ ^]^ Natural forests generally have higher soil microbial carbon use efficiency than plantations,^[^ ^38^ ^]^ and a higher microbial carbon use efficiency is found to improve SOC by accumulating microbial‐derived carbon.^[^ ^39^, ^40^ ^]^ In addition, natural forests have higher plant diversity than plantations, which promotes SOC directly by increasing carbon inputs from plants^[^ ^41^ ^]^ or indirectly by improving microbial carbon use efficiency and microbial‐derived carbon.^[^ ^42^, ^43^, ^44^ ^]^ Plant diversity also increases the molecular diversity of SOC, which is found to increase the metabolic demand of, and thus potentially limit, decomposition.^[^ ^45^, ^46^ ^]^

The random forest models showed that mean annual temperature (MAT) and aridity index (AI, a greater AI indicates a humid climate) were the most important drivers of both MAOC and POC, supporting the negative effects of temperature and water stress on MAOC and POC (Figure 2a,b). Importantly, the interaction coefficient between MAT and forest type (for plantation) in the linear mixed‐effect models was negative (Figure 2c,d; Figures S1 and S2, Supporting Information), suggesting that climate warming accelerates soil carbon loss during the conversion of natural forests to plantations (Figure 2e,f). Conversely, the interaction coefficient between AI and forest type (for plantation) was positive (Figure 2c,d; Figures S1 and S2, Supporting Information), indicating that climate drying exacerbates soil carbon loss during this conversion process. First, the effect of plant diversity on SOC increases with increasing plant richness,^[^ ^47^, ^48^ ^]^ and tree species diversity also reduces SOC loss by decreasing the temperature sensitivity of SOC decomposition.^[^ ^49^ ^]^ On one hand, the natural forests in warm regions have significantly higher diversity than those in cold regions; one the other hand, tree diversity effect on SOC itsself is stronger in warm regions than in cold regions.^[^ ^44^ ^]^ Therefore, it is expected that the diversity effects of the former regions on SOC are stronger than that of the latter regions. Second, the productivity of natural forests is more resistant to water stress than plantations.^[^ ^50^, ^51^ ^]^ Especially, the same tree species may exhibit completely contrasting vulnerability and resilience to drought‐induced decline in productivity, with a greater vulnerability and lower resilience in plantations.^[^ ^52^ ^]^ Therefore, converting natural forests to plantations would result in greater MAOC and POC losses in dry regions compared to humid regions, as the conversion leads to a more pronounced decline in aboveground productivity in dry areas than in humid ones. In addition, a huge disaster of German plantations triggered by climate change has ignited a fierce debate over how the nation should manage its trees, which further supports the higher vulnerability of plantations in response to climate change.^[^ ^53^ ^]^ In this context, our findings indirectly suggest that MAOC and POC in plantations may be more sensitive to climate warming and drying than those in natural forests.

### 2.2 The Deficits of Soil MAOC and POC in Plantations Compared with Natural Forests

**Figure 3:**
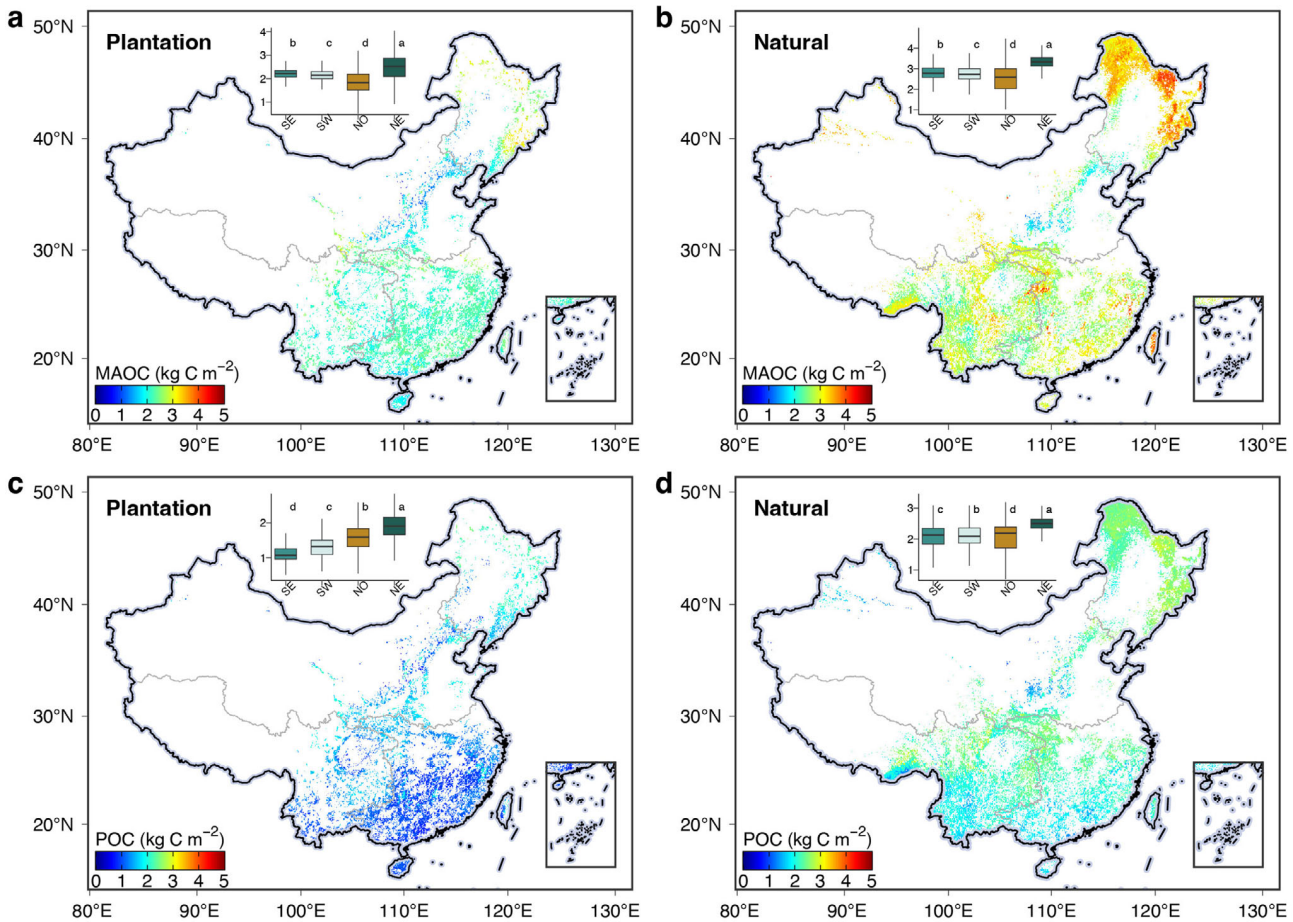
Distributions of soil functional organic carbon fractions in China's forest. a,b) Spatial distribution of mineral‐associated organic carbon (MAOC) in planted forests (left) and natural forests (right). c,d) Distribution of particulate organic carbon (POC) in plantations (left) and natural forests (right). The letters indicate different levels among different regions (*p* < 0.05; one‐way analysis of variance). SE, Southeast China; SW, Southwest China; NO, Northern China; NE, Northeast China.

**Figure 4:**
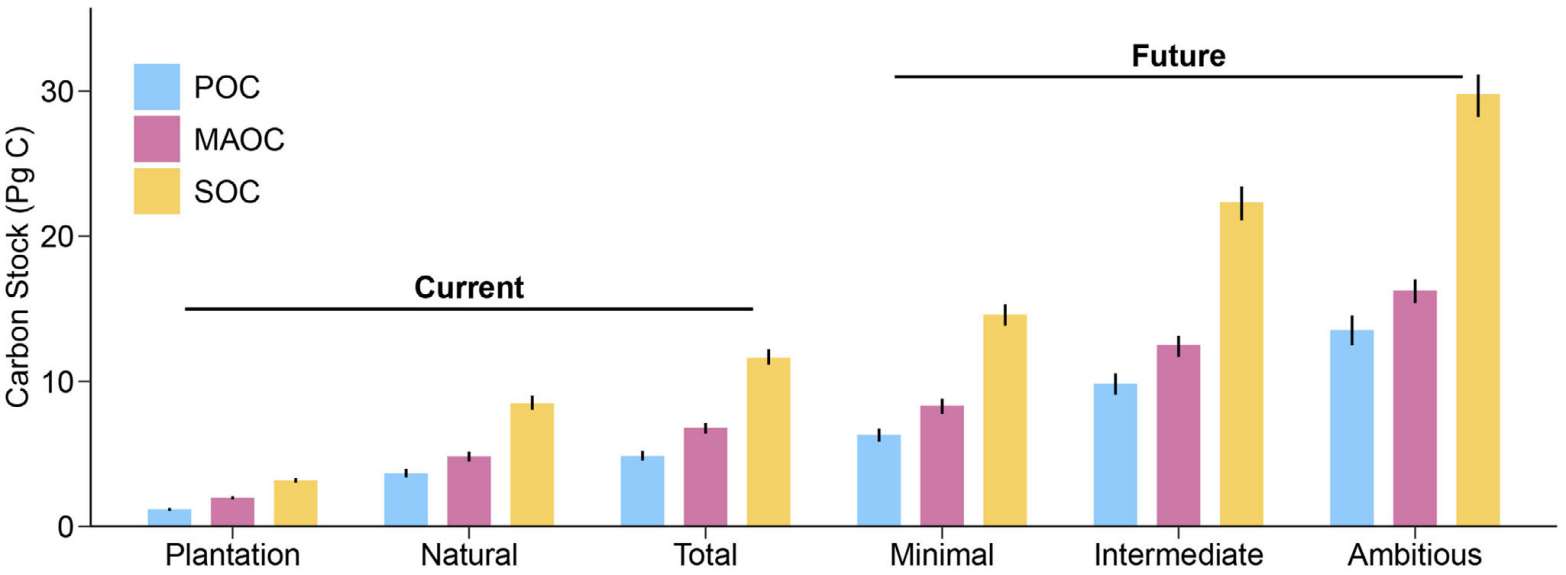
Current and potential forest soil carbon storage. The 60% percentile (a quantile random forest model) was regarded as a minimal objective, the 80% percentile was regarded as an intermediate objective, while the 90% percentile was regarded as an ambitious objective. Error bars represent the 95% confidence intervals derived from 100‐time bootstrapping. POC, particulate organic carbon; MAOC, mineral‐associated organic carbon; SOC, soil organic carbon.

Forest type (plantations vs natural forests), climates (MAT and AI), soil properties (soil pH and clay plus silt content), net primary productivity, and elevation together explained 77% and 75% of the spatial variations of MAOC and POC, respectively (random forest models, **Figure** **3**a,d). The tenfold cross‐validation further suggested that our random forest models had good performance in predicting the spatial variations of both MAOC (*R* ^2^ = 0.77, Figure S3, Supporting Information) and POC (*R* ^2^ = 0.75, Figure S3, Supporting Information). Expanding these high‐precision random forest models to a national scale, we generated the topsoil MAOC and POC maps and derived MAOC and POC storage of $4.82_{4.49}^{5.15}$ Pg C (mean with 5% and 95% quantile) and $3.66_{3.31}^{3.95}$ Pg C in China's natural forests, and $1.97_{1.87}^{2.09}$ Pg C and $1.18_{1.08}^{1.28}$ Pg C in China's plantations, respectively (Figures 3 and **4**). The total forest topsoil SOC storage was $11.63_{11.16}^{12.23}$ Pg C, which was well consistent with previous estimations ranging from 8.49 to 13.93 Pg C.^[^ ^54^, ^55^, ^56^ ^]^

**Figure 5:**
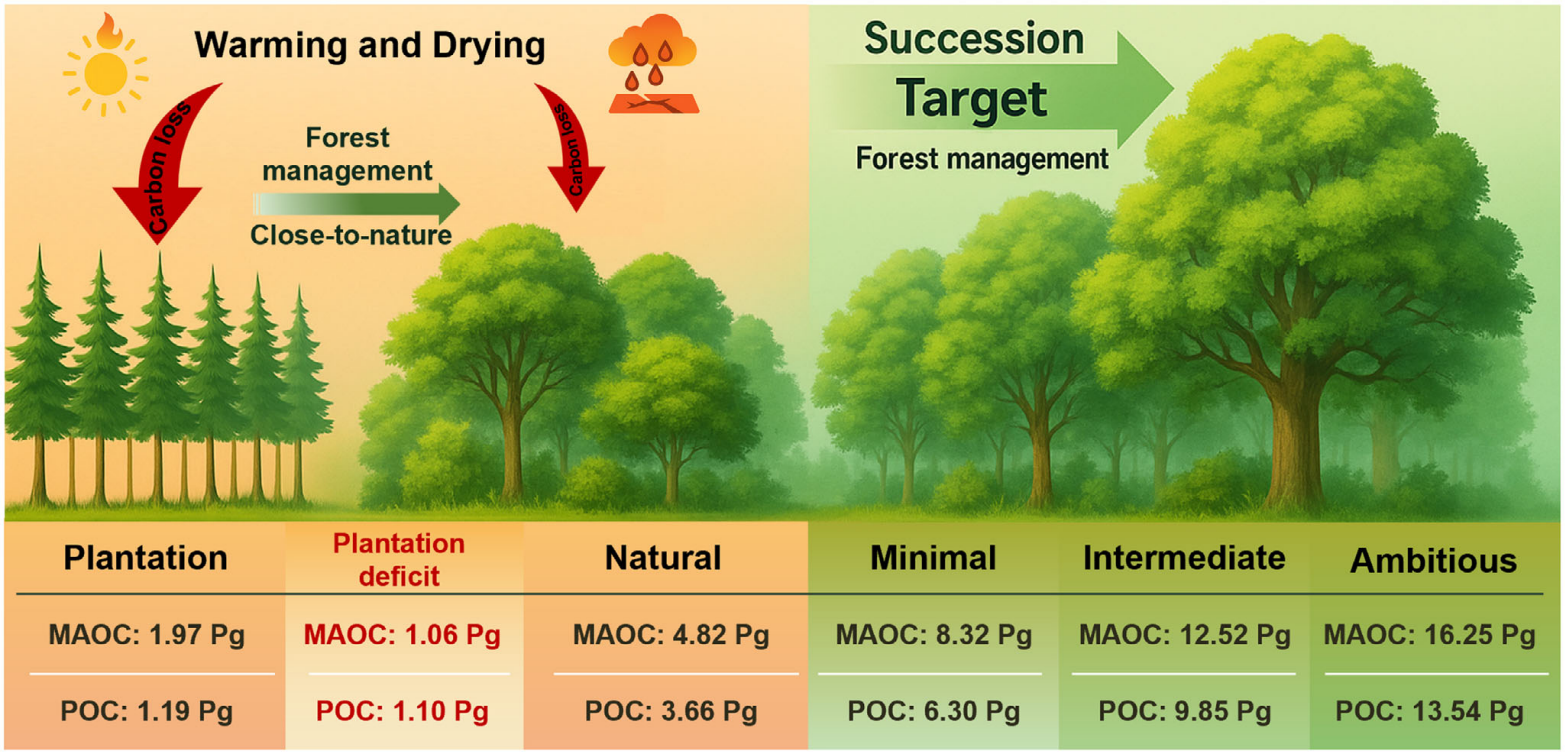
Conceptual diagram illustrating the storage potential of soil functional carbon fractions in China's forests. The minimal, intermediate, and ambitious objectives correspond to the 60%, 80%, and 90% percentiles (quantile random forest models), respectively. POC, particulate organic carbon; MAOC, mineral‐associated organic carbon.

The random forest models were also used to estimate the deficits of soil MAOC and POC in plantations compared to natural forests. We obtained the total deficit of SOC by $2.17_{1.87}^{2.52}$ Pg C, with MAOC contributing $1.06_{0.88}^{1.26}$ Pg C and POC contributing $1.10_{0.91}^{1.31}$ Pg C (**Figure** **5**). Plantations in southeast and southwest China had greater deficits of MAOC and POC stocks than northeast and north China (Figure S4, Supporting Information) because converting natural forests to plantations would result in greater MAOC and POC losses in warm regions compared to cold regions (the above discussion). The net carbon sink of terrestrial ecosystems in China ranged from 0.19 to 0.26 Pg C per year.^[^ ^57^ ^]^ Therefore, if soil MAOC and POC in plantations reach the levels of natural forests, which could represent about ten‐year accumulative land carbon sink in China. In addition, the French Minister of Agriculture Stéphane Le Foll at COP21 set the “soil carbon 4 per mille”, i.e., increasing SOC stocks by 0.4 percent per year as compensation for the global emissions of greenhouse gases by anthropogenic sources.^[^ ^58^ ^]^ The estimated deficits of plantation SOC storage compared to natural forests were up to 17% of total forest topsoil SOC storage, representing about 40 years’ soil carbon 4 per mille. We believe that this time window is sufficient to manage the plantations to eliminate the current SOC deficit. For example, close‐to‐nature management of plantations was suggested to be an effective pathway for achieving carbon neutrality within this period.^[^ ^59^ ^]^ In specific, we conducted a global meta‐analysis of the effects of close‐to‐nature management of plantations on SOC and showed that these effects increased with the duration of management (Figure S5, Supporting Information). If the duration of close‐to‐nature management is greater than 15 years, the SOC would be increased by 43%. Therefore, the observed deficit of plantations SOC storage compared to natural forests can be gained by sustainable plantation management.

### 2.3 MAOC and POC Storage Potential Across China's Forests

The estimations of SOC storage potential reflect the maximum attainable potential (i.e., maximum effective capacity) of SOC stocks under these different pedoclimatic conditions, which would help to develop the optimal forest management practices to improve SOC storage.^[^ ^60^, ^61^ ^]^ To estimate such SOC potential, a quantile random forest model (see Methods) was used to quantify the maximum SOC storage potential (Figure 4; Figure S6, Supporting Information). It is expected that the SOC storage potential is very sensitive to the percentile used in the calculation. In specific, a minimal objective (60% percentile) of SOC storage potential was $14.61_{13.86}^{15.31}$ Pg C in topsoil, with MAOC and POC potentials of $8.32_{7.76}^{8.80}$ Pg C and $6.3_{5.83}^{6.73}$ Pg C; an intermediate objective (80% percentile) of SOC storage potential was $22.36_{21.12}^{23.43}$ Pg C in topsoil, with MAOC and POC potential of $12.52_{11.68}^{13.15}$ Pg C and $9.85_{9.09}^{10.55}$ Pg C; and an ambitious objective (90% percentile) of SOC storage potential was $29.79_{28.25}^{31.15}$ Pg C in topsoil, with MAOC and POC potentials of $16.25_{15.42}^{17.02}$ Pg C and $13.54_{12.49}^{14.54}$ Pg C, respectively.

We also explored the factors that constrain the additional carbon stocks (the difference between current carbon stocks and potential carbon stocks), and found that MAT was the dominant factor controlling the spatial distribution of additional MAOC and POC storage (*R* ^2^ = 0.67–0.86; Figure S7, Supporting Information). These findings also support the view that maximum attainable potential is constrained by the particular climatic conditions.^[^ ^61^ ^]^ Partial dependence analyses further showed that colder zones tend to exhibit higher additional MAOC and POC potential (Figure S8, Supporting Information), implying that climate warming would reduce the additional carbon storage.

There are several limitations and uncertainties that warrant further consideration. First, forest subsoil (>20 cm) SOC accounts for half of total SOC storage relative to the first‐meter soils.^[^ ^62^ ^]^ Subsoil SOC is equally or even more sensitive to climate warming;^[^ ^63^, ^64^, ^65^ ^]^ however, data on MAOC and POC in subsoils remain extremely limited for China's forests. Second, although our models exhibited high predictive accuracy, they did not incorporate partial ecological factors such as tree species composition, forest age, and biodiversity due to the lack of comprehensive datasets. Mixed‐species forests generally store more SOC than monoculture plantations, while forest age and biodiversity influence SOC accumulation by modulating plant inputs and microbial processes.^[^ ^66^ ^]^ Third, SOC has a long turnover time; the current SOC storage is influenced by land use types beyond the current forest type.^[^ ^67^ ^]^ Therefore, historical land‐use legacies may influence the accuracy of our assessments. Fourth, our estimation of the influence of forest management and climate on MAOC and POC is based on the space‐for‐time approach, which may introduce uncertainty, despite its widespread application.^[^ ^68^, ^69^ ^]^ Fifth, we discussed the effects of close‐to‐nature management of plantations on SOC using a mini meta‐analysis. While natural forest management also has the potential to restore soil carbon,^[^ ^70^ ^]^ its contribution to SOC potential requires further experimental validation. Finally, the inconsistent experimental procedures, like inconsistent sampling time and soil depth, also introduce uncertainties for our estimations.

In summary, our study presents the first comprehensive national‐scale assessment of topsoil (0–20 cm) MAOC and POC stocks in China's natural and planted forests. Our estimations of MAOC and POC stocks, derived using the random forest method, are $4.82_{4.49}^{5.15}$ Pg C and $3.66_{3.31}^{3.95}$ Pg C in natural forests, and $1.97_{1.87}^{2.09}$ Pg C and $1.18_{1.08}^{1.28}$ Pg C in plantations, respectively. The total deficit of SOC in plantations compared with natural forests is $2.17_{1.87}^{2.52}$ Pg C. The meta‐analysis further demonstrates that close‐to‐nature management practices in plantations can enhance SOC storage by 43%, provided the management duration exceeds 15 years. Thus, this timeframe is sufficient to manage plantations and eliminate the current SOC deficit. Additionally, the potential for MAOC and POC storage can serve as a key target for forest management to support global carbon neutrality goals.

## 3 Experimental Section

### Forest Soil Survey across China

During the growing seasons of 2021 and 2022, the study sampled 248 forest cases across China, including 165 natural forests and 83 planted forests (Figure 1a). These cases spanned a broad geographic and climatic gradient, with longitude ranging from 81.17°E to 132.07°E, latitude from 21.64°N to 53.33°N, mean annual temperature from −4.73 to 21.01 °C, and mean annual precipitation from 190 to 1791 mm. At each sample, three 20 m × 20 m plots were established, with each plot spaced more than 30 m. Within each plot, ten locations were randomly selected to collect topsoil samples (0–10 cm in 2021 and 0–20 cm in 2022), which were then combined to form a composite sample for each plot. All samples were sieved (<2 mm) to remove gravel and coarse fragments, and visible roots and plant debris were removed for further analysis. The elevation and soil type were also recorded at each site.

In addition, databases of Web of Science, Google Scholar, and China National Knowledge Infrastructure were searched using the keywords “mineral‐associated organic carbon”, “particulate organic carbon”, “MAOC”, “POC”, and “soil carbon fraction” along with “forest”. For papers that reported soil data across different seasons and sampling times, the study calculated mean values for each forest. In studies reporting values for individual replicates within a site, the mean for each study site was also calculated. For global change experiments, only the data from the control group were included. Combining these data points with the national‐scale survey, a final dataset comprising 139 studies with 399 observations from natural forests and 418 from plantations was compiled.

### Soil Characterization and Carbon Fractionation

Soil pH was measured in a 1:2.5 soil‐to‐deionized water mixture using a pH electrode (FE28‐Standard, Mettler, Switzerland), and soil texture was determined with a Malvern laser particle size analyzer (MS2000; Malvern Instruments, Malvern, UK). Physical fractionation techniques were used to distinguish mineral‐associated organic matter (MAOM) and particulate organic matter (POM) components. In specific, the air‐dried soil samples were mixed with a 0.5% sodium hexametaphosphate solution at a ratio of 1:3 (soil to solution, w/v) and agitated for 18 hours at room temperature. Following dispersion, the soil mixture was passed through a 270 mesh (53 µm) sieve and rinsed with water until the effluent was clear. The material retained on the sieve was collected as POM, while the portion that passed through was collected as MAOM.^[^ ^14^ ^]^ The carbon concentrations in MAOM and POM were analyzed using a Multi C/N 2100s analyzer (Analytik Jena AG, Germany) after the removal of inorganic carbon by hydrochloric acid. Finally, the study also calculated the means across three plots in each study site for the following analyses.

### Environmental Covariates and Data Sources

Factors influencing the spatial distributions of MAOC and POC included soil pH, clay plus silt content, elevation, soil type, net primary productivity, mean annual temperature, and aridity index (ratio of precipitation to potential evapotranspiration). If mean annual temperature was unavailable in the original studies, it was extracted from WorldClim 2.^[^ ^71^ ^]^ The aridity index was obtained from the Global Aridity Index and Potential Evapotranspiration Climate database.^[^ ^72^ ^]^ Elevation was sourced from OpenLandMap (https://openlandmap.org). If edaphic factors (clay plus silt content, and pH) were missing, they were retrieved from the China dataset of soil properties for land surface modelling version 2 (CSDLv2), which effectively represents the spatial variation of soil properties across China.^[^ ^73^ ^]^ The soil order was obtained from the Harmonized World Soil Database version 2 (HWSD 2.0).^[^ ^74^ ^]^ Additionally, the mineral type was classified by soil type according to Georgiou et al.^[^ ^68^ ^]^: Oxisols and Ultisols were classified as low‐activity minerals, while Alfisols, Aridisols, Andisols, Entisols, Gelisols, Inceptisols, Mollisols, Spodosols, and Vertisols were categorized as high‐activity minerals. Net primary productivity was derived from Moderate Resolution Imaging Spectroradiometer,^[^ ^75^ ^]^ representing the mean net primary productivity across 2001–2023.

### Meta‐Analysis of Close‐To‐Nature Management

The objective of close‐to‐nature management is to restore planted forests to a near‐natural state.^[^ ^76^ ^]^ The predominant approach involved establishing age‐diverse mixed‐species forests to enhance stand structure and improve vegetation productivity, which in turn promotes SOC sequestration.^[^ ^77^, ^78^ ^]^ A literature search across Web of Science, Google Scholar, and China National Knowledge Infrastructure was conducted using keywords such as (“close‐to‐nature management”, “interplant” or “intercrop”) along with (“organic carbon” or “organic matter”) to gather peer‐reviewed articles assessing the impact of close‐to‐nature management on SOC content. Additionally, papers needed to specify both the duration of the close‐to‐nature transformation and the sampling depth. The final dataset included 34 studies with a total of 83 topsoil (<20 cm) independent observations.

The natural log‐transformed response ratio (*RR*)^[^ ^79^ ^]^ was employed to quantify the effects of close‐to‐nature management on SOC change:

(1) $$RR=\ln\left(\frac{x_{t}}{x_{c}}\right)=\ln\left(x_{t}\right)\;-\ln\left(x_{c}\right)$$

where *x*_t_ and *x*_c_ represent the mean values of SOC in the management and control treatments, respectively.

The variance (*v*) of RR was calculated as:

(2) $$v=\frac{S_{t}^{2}}{n_{t}x_{t}^{2}}+\frac{S_{c}^{2}}{n_{c}x_{c}^{2}}$$

where *n*_t_ and *n*_c_ denote the number of replicates for the control and treatment, *S*_t_ and *S*_c_ are the corresponding standard deviations. If the standard deviation was not reported, it was estimated as 1/10 of the mean.^[^ ^80^, ^81^ ^]^

A random‐effect model was applied to evaluate the impact of close‐to‐nature management on SOC using the “metafor” package.^[^ ^82^ ^]^

### Statistical Analysis

Mixed‐effects models were conducted via “lme4” and “lmerTest” packages^[^ ^83^, ^84^ ^]^ to quantify the effects of forest types (plantations vs natural forests) and environmental factors on natural‐log‐transformed MAOC and POC:

(3) $$MAOC\;or\;POC=\beta_{0}+\beta_{1}E+\beta_{2}T+\beta_{3}\left(T\times E\right)+\pi_{study}+\varepsilon$$

where *E* represents environmental variables, including soil pH, clay plus silt content, elevation, soil type, net primary productivity, mean annual temperature, and aridity index; *T* represents forest types (plantations vs natural forests); π_*study*_ represents the random effect of study; ε is the error. All predictors were standardized prior to model fitting for comparability across variables. Model selection was performed using backward elimination based on the Akaike Information Criterion. Ultimately, interaction terms with pH and clay plus silt content were excluded from the MAOC model, while elevation with its interaction terms and the net primary productivity interaction were excluded to yield the final POC model (Figure S1, Supporting Information).

A workflow to predict the storage potential of MAOC and POC for China's forests (Figure S5, Supporting Information) was then proposed. Specifically, the performances of five widely used machine learning algorithms (random forest, support vector machine, multivariable linear regression, neural network, and regression trees) were first assessed by employing tenfold cross‐validation using predictors of mean annual temperature, aridity index, net primary productivity, elevation, clay plus silt content, pH, sample depth, mineral type, and forest type (plantations vs natural forests). The results indicated that the random forest exhibited the best performance, yielding the lowest root mean square error for predicting both MAOC and POC (Table S1 and Figure S3, Supporting Information). Sensitivity analysis showed that the choice of 1000 trees ensured model performance and robustness (Figure S9, Supporting Information). Furthermore, the study utilized the “permutation” feature importance method from “ranger” package^[^ ^85^ ^]^ to evaluate the importance of each predictor.

To evaluate spatial autocorrelation in the model outputs, semivariograms of the MAOC and POC concentrations were calculated, which did not exhibit strong spatial autocorrelation patterns. The study then calculated Moran's I on the residuals of the random forest models to statistically assess spatial dependence. A significantly positive Moran's I would indicate spatial clustering in model prediction errors. Here, the results showed no significant spatial autocorrelation in either the MAOC or POC models (Figure S10, Supporting Information).

Next, the MAOC and POC maps were generated by bootstrapping approach. In specific, all predictors were resampled to a 1 km × 1 km resolution. The 100‐time bootstrapping was used to generate the uncertainties of global maps (for each bootstrapping, 70% of the data were randomly sampled). The MAOC and POC stocks were calculated as:

(4) $$SOCS=SOCC\times BD\times \left(1-F\right)\times D$$

where *SOCS* is SOC stocks, *SOCC* is SOC concentration. *BD* is bulk density, *F* is the volumetric proportion of coarse fraction, and *D* is soil depth. Both bulk density and the volumetric proportion of coarse fraction were obtained from CSDLv2.^[^ ^73^ ^]^

Subsequently, the study estimated the deficits of MAOC and POC in plantations compared to natural forests by setting forest types as natural forests in the random forest models. The same 100‐time bootstrapping was used to assess uncertainty in the estimated deficits (Figure S11, Supporting Information).

Finally, quantile random forest models were employed using the “ranger” package,^[^ ^85^ ^]^ which excels at handling multivariate nonlinear relationships and high‐dimensional data, to establish SOC maximum attainable potential.^[^ ^61^, ^86^ ^]^ Referring to previous studies on SOC potential assessment,^[^ ^21^, ^61^, ^86^, ^87^, ^88^ ^]^ the study defined three quantile‐based scenario objectives: the 60% percentile represents a minimal objective under moderate management, the 80% percentile represents an intermediate objective under favorable conditions or good management, and the 90% percentile reflects an ambitious objective approaching the maximum attainable potentials of soils. In these quantile random forest models, the forest type was set as natural forest because natural forests had greater MAOC and POC storage than plantations. All statistical analyses were conducted in R 4.4.2 (R Core Team 2024).

## Conflict of Interest

The authors declare no conflict of interest.

## Author Contributions

Z.Z. and S.L. conceived and designed the project. T.Z. and Z.Z. conducted the field sampling. S.R. collected and analyzed the data. S.R. and Z.Z. wrote the initial draft. All authors contributed to discussing the data analyses and results and writing and editing the paper.

## Supporting information



Supporting Information

Supporting Information

## Data Availability

The data that support the findings of this study are available in the supplementary material of this article.
