# Supplementary material for: Storage Potential of Soil Functional Carbon Fractions in the World's Largest Plantations
Source: Adv Sci (Weinh). 2025 Aug 7;12(41):e04995. doi: 10.1002/advs.202504995 (PMC12591171; doi:10.1002/advs.202504995)
Supplement: Supplementary file 1 — Supporting Information [file ADVS-12-e04995-s002.docx]

Supporting Information

Storage Potential of Soil Functional Carbon Fractions in the World's Largest Plantations

Siyu Ren^1,2^, Hui Wang^3^, Tao Zhou^1,2^, Chuankuan Wang^1,2^, Zhenghu Zhou^1,2,^*, Shirong Liu^3,^*

**Figure S1.** Linear mixed-effects model of mineral-associated organic carbon (MAOC) and particulate organic carbon (POC) after model selection. The panels display (dots) and 95% confidence intervals (CI, error bars) for the main effects and interactions of forest type and environmental factors, collectively explaining 79% and 74% of the total variance for MAOC and POC, respectively (*n* = 1065 for MAOC and POC observations). MAT, mean annual temperature; AI, aridity index; CS, clay plus silt content; NPP, net primary productivity; Type, type of plantation (plantations versus natural forests).

**
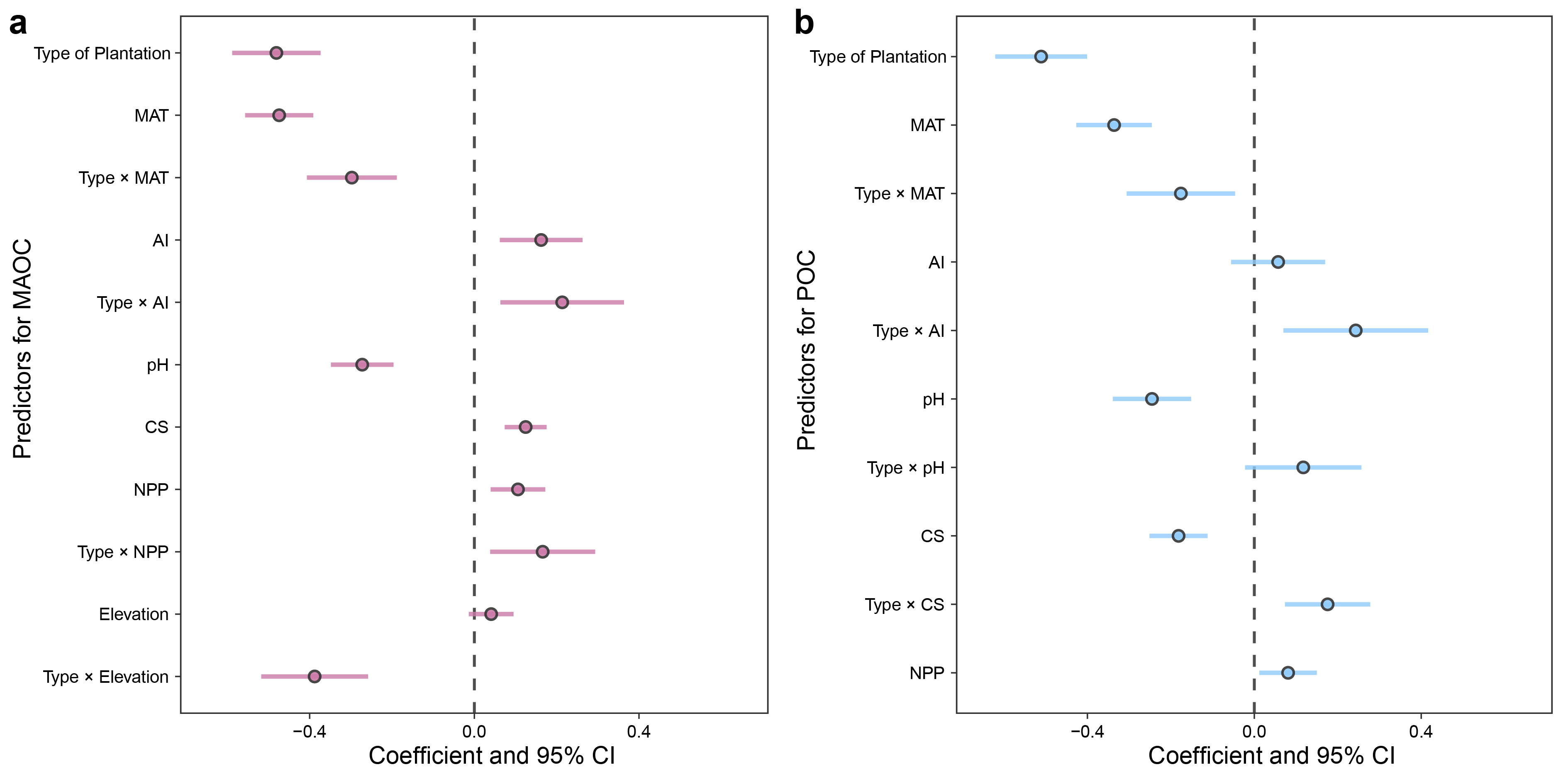
**

**Figure S2. Effects of environmental factors on mineral-associated organic carbon (MAOC) and particulate organic carbon (POC) contents in planted and natural forests.** Standardized (dots) and 95% confidence intervals (CI, error bars) from linear mixed-effects models showing the influence of environmental predictors on a) MAOC and b) POC. Separate models were fitted for plantation and natural forests. Fixed and random effects together explained 89% (MAOC) and 77% (POC) of the variance in plantations, and 64% (MAOC) and 60% (POC) in natural forests (*n* = 501 for plantations, *n* = 564 for natural forests). MAT, mean annual temperature; AI, aridity index; NPP, net primary productivity.

**
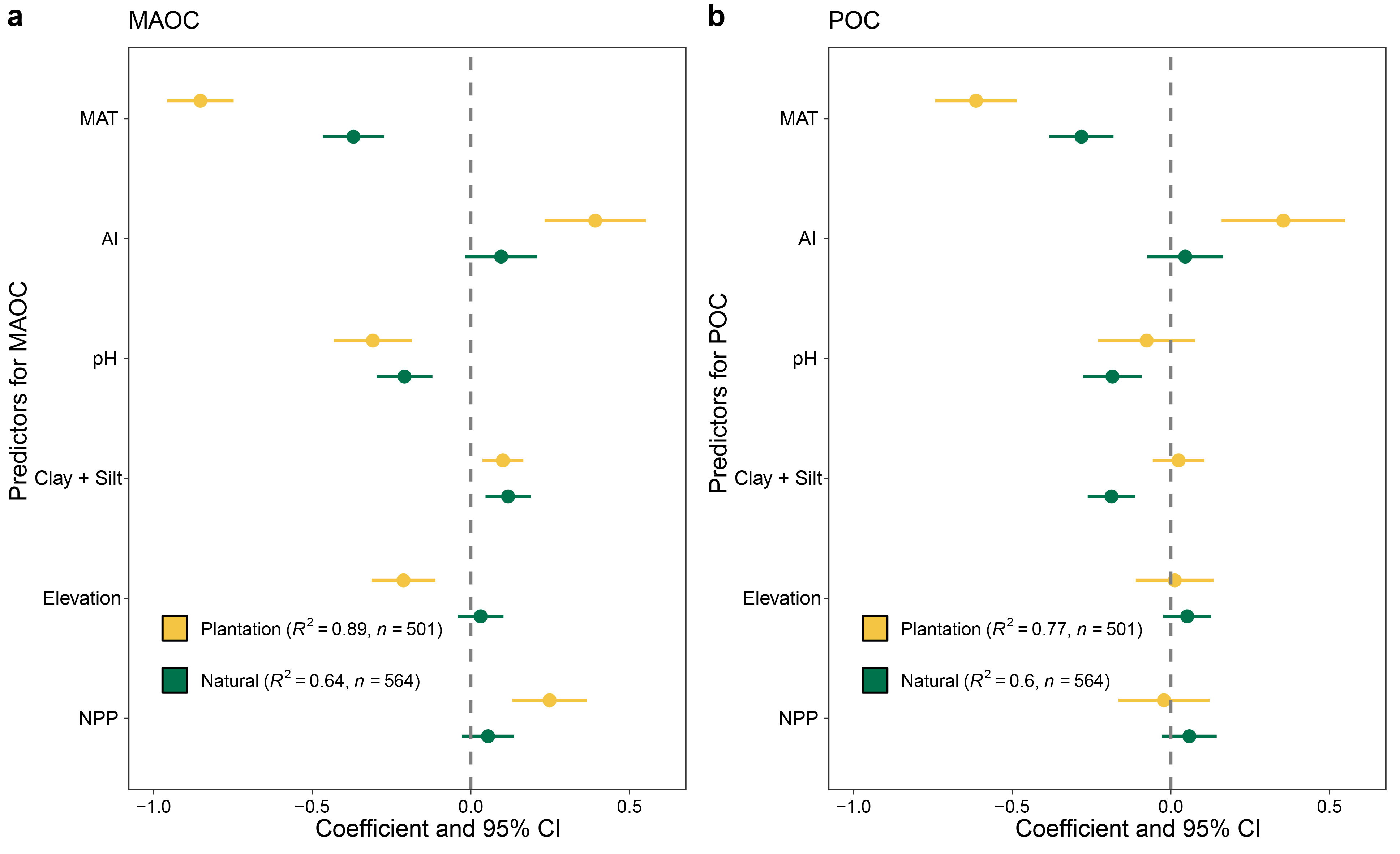
**

**Figure S3.** The validation of random forest models. MAOC, mineral-associated organic carbon; POC, particulate organic carbon.

**
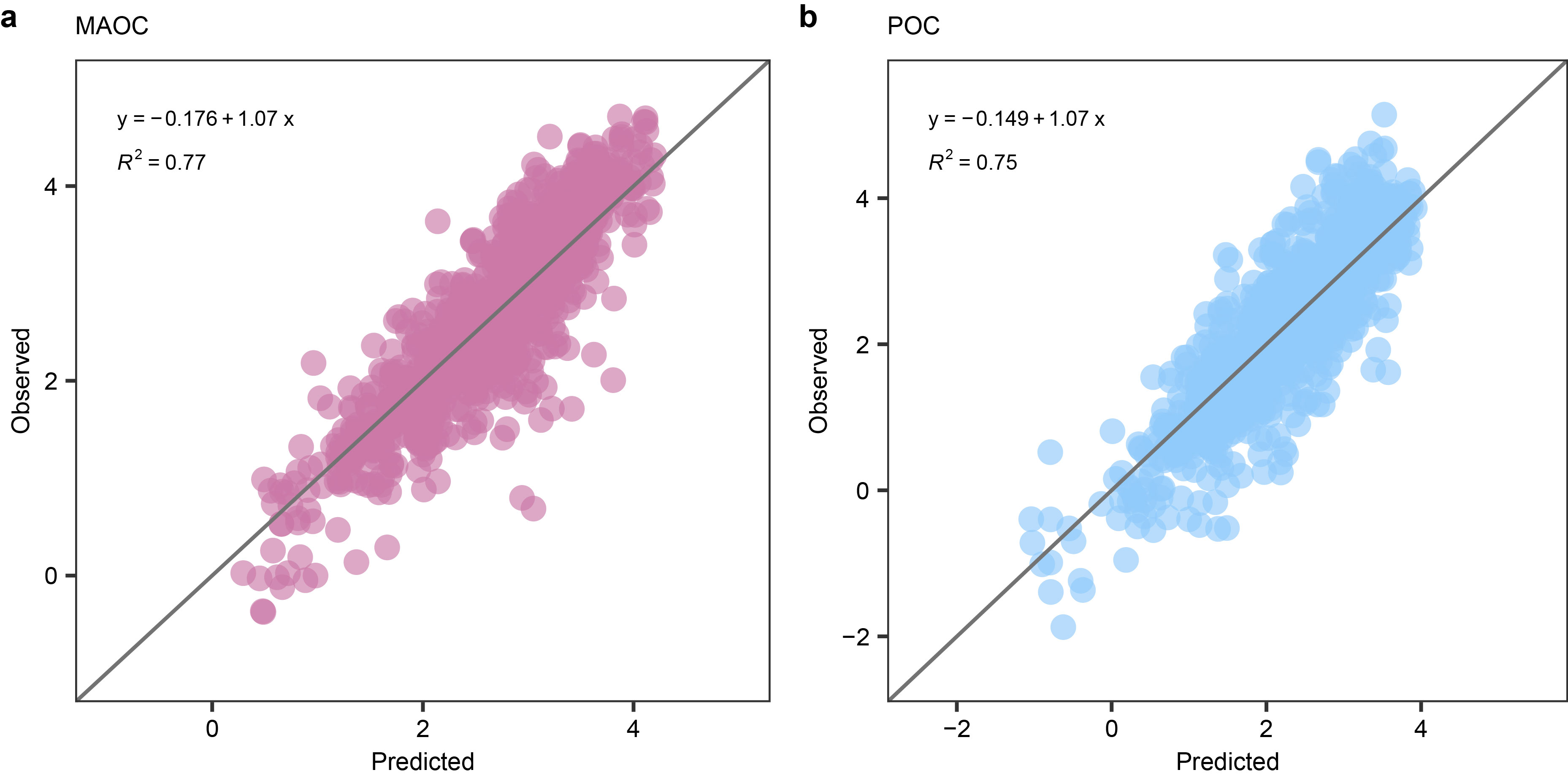
**

**Figure S4.** The deficit of mineral-associated and particulate organic carbon in plantations compared with natural forests. a), Mineral-associated organic carbon (MAOC) and b), particulate organic carbon (POC) deficit in planted forests. SE, Southeast China; SW, Southwest China; NO, Northern China; NE, Northeast China.

**
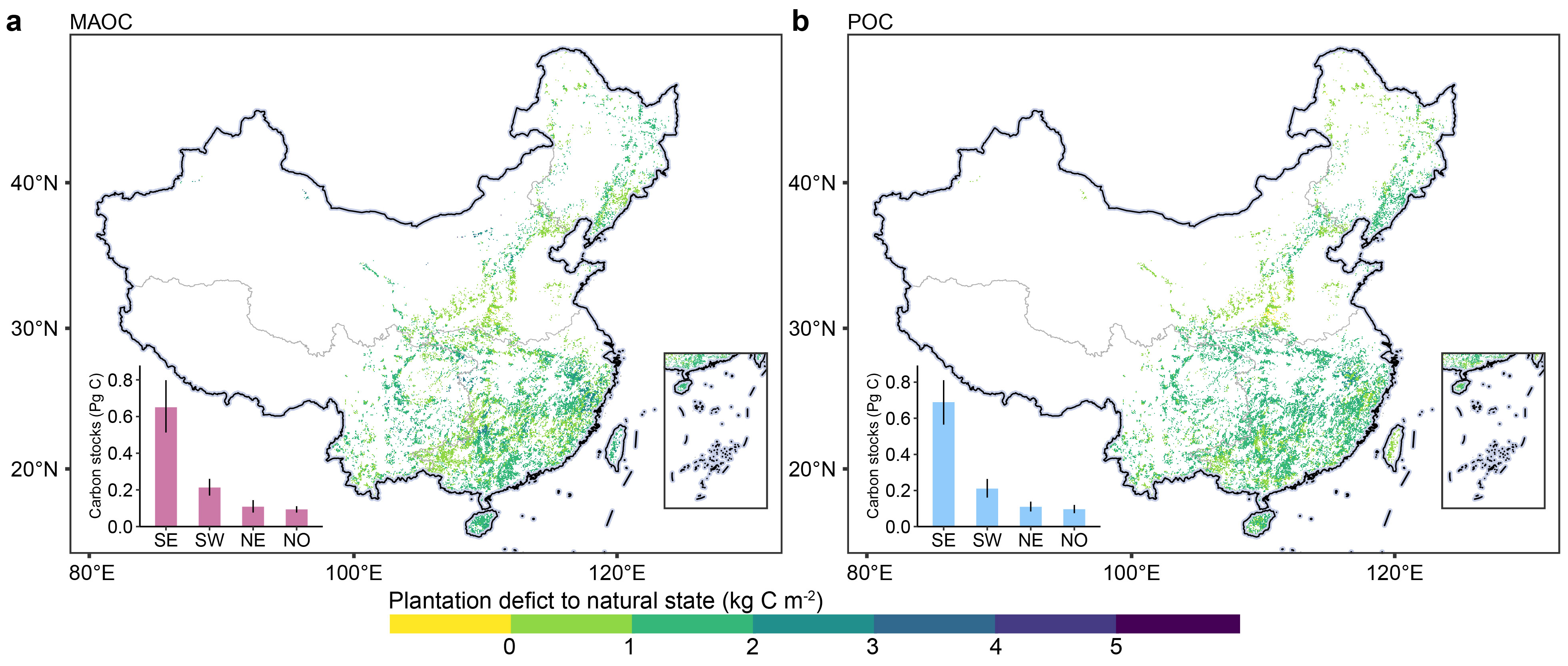
**

**Figure S5.** Percentage change in soil organic carbon via close-to-nature management. a), Total effects, b) management years and c) tree species on soil organic carbon change. Error bars represent the 95% confidence intervals. The numbers *n* in the figures are the corresponding number of observations. d), Distribution of effect values with mean annual temperature (MAT).

**
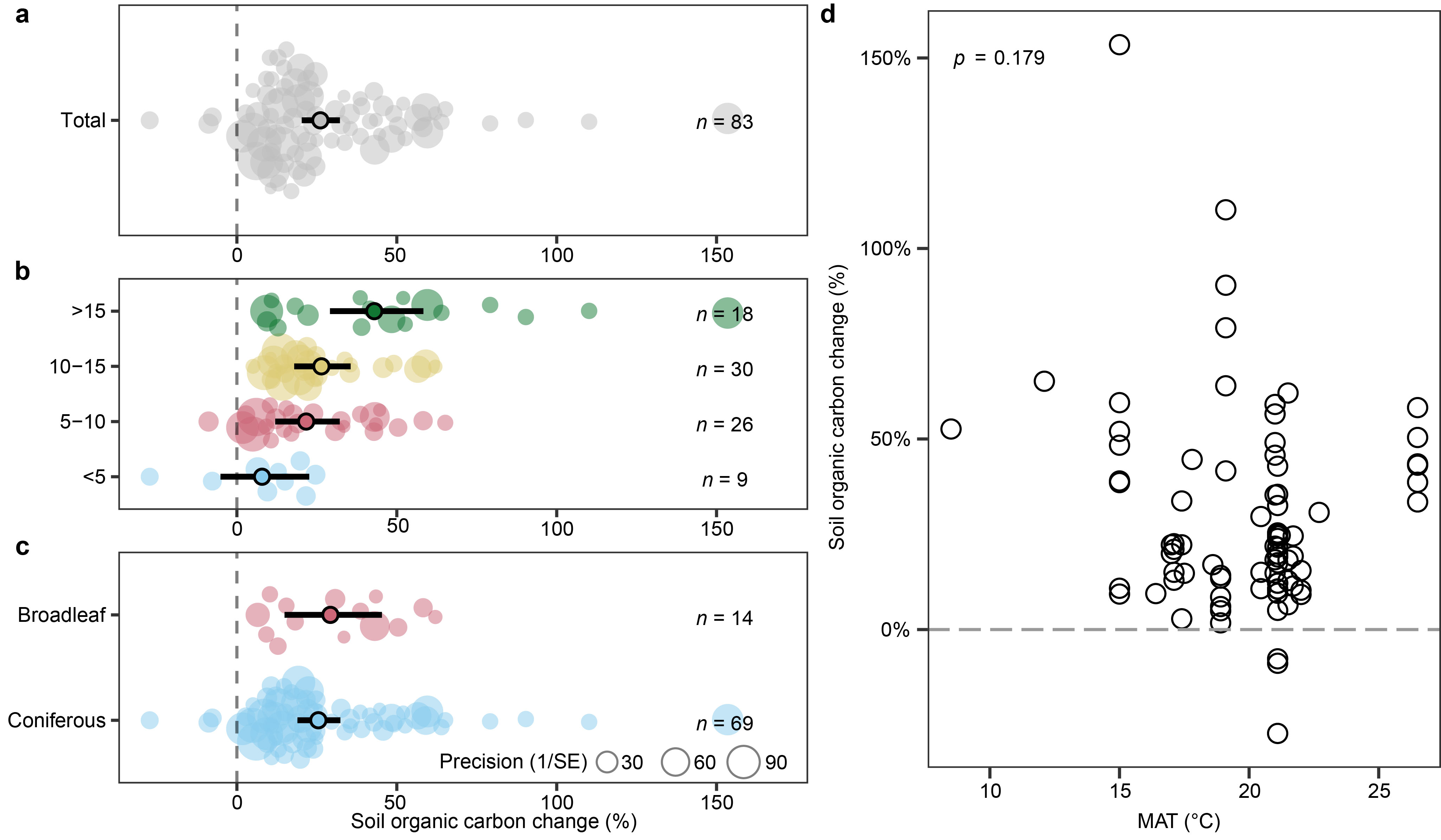
**

**Figure S6.** Distributions of storage potential of mineral-associated and particulate organic carbon in China's forests. The (a, b) 60% percentile (a quantile random forest model) was regarded as a minimal objective, the (c, d) 80% percentile was regarded as an intermediate objective, and the (e, f) 90% percentile was regarded as an ambitious objective. MAOC, mineral-associated organic carbon; POC, particulate organic carbon.

**
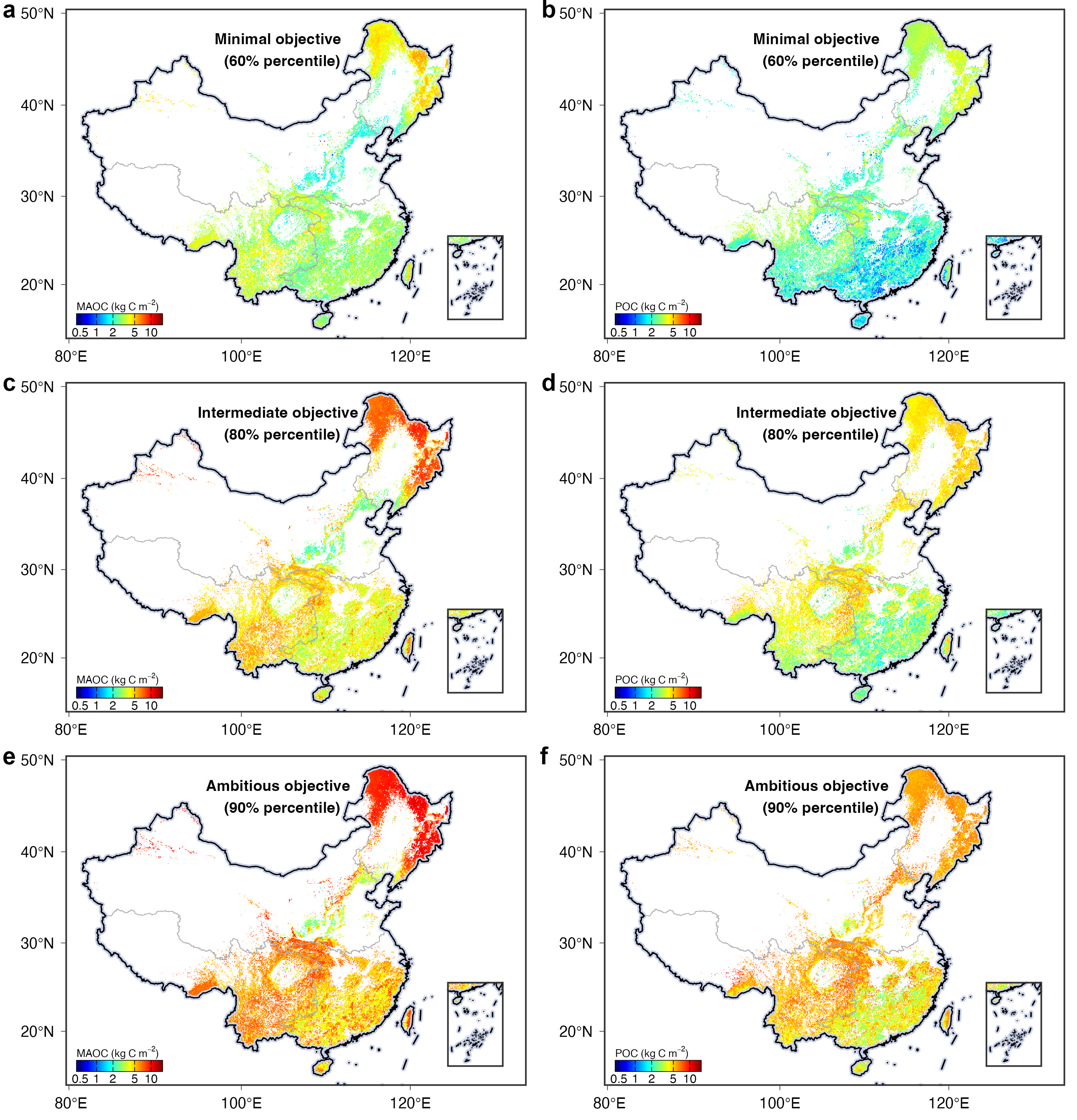
**

**Figure S7.** Variable importance for predicting the difference between current carbon stocks and potential carbon stocks of mineral-associated organic carbon (MAOC; a–c) and particulate organic carbon (POC; d–f) using random forest models. MAT, mean annual temperature; AI, aridity index; NPP, net primary productivity; CS, clay plus silt content.

**
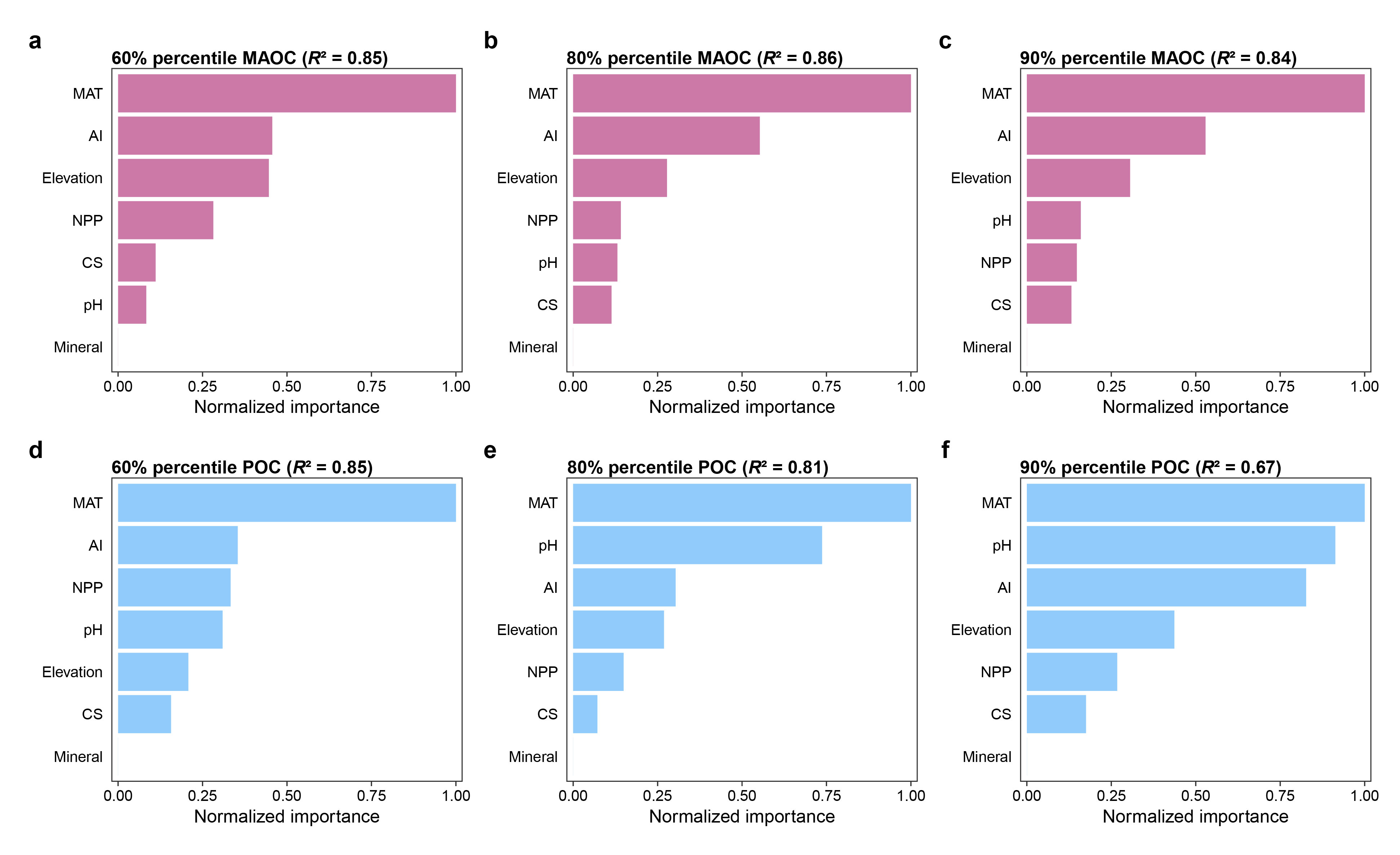
**

**Figure S8.** Partial dependence of environmental predictors on mineral-associated organic carbon (MAOC) and particulate organic carbon (POC) additional stocks across different quantiles. MAT, mean annual temperature; AI, aridity index; NPP, net primary productivity.

**
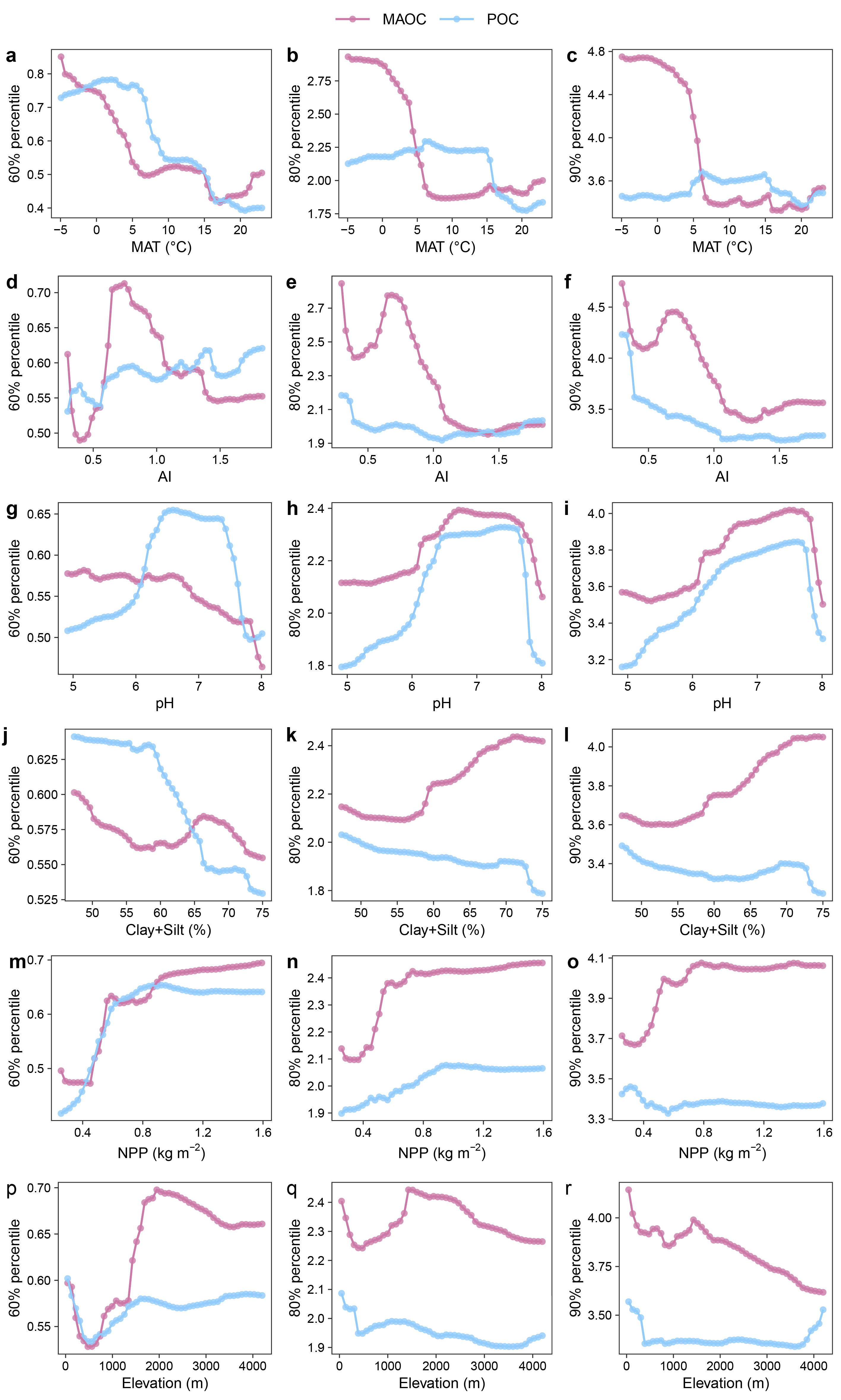
**

**Figure S9.** Sensitivity of random forest model performance to the number of trees. a) R squared (*R*²) and b) mean squared error (MSE) for mineral-associated organic carbon (MAOC) and particulate organic carbon (POC).

**
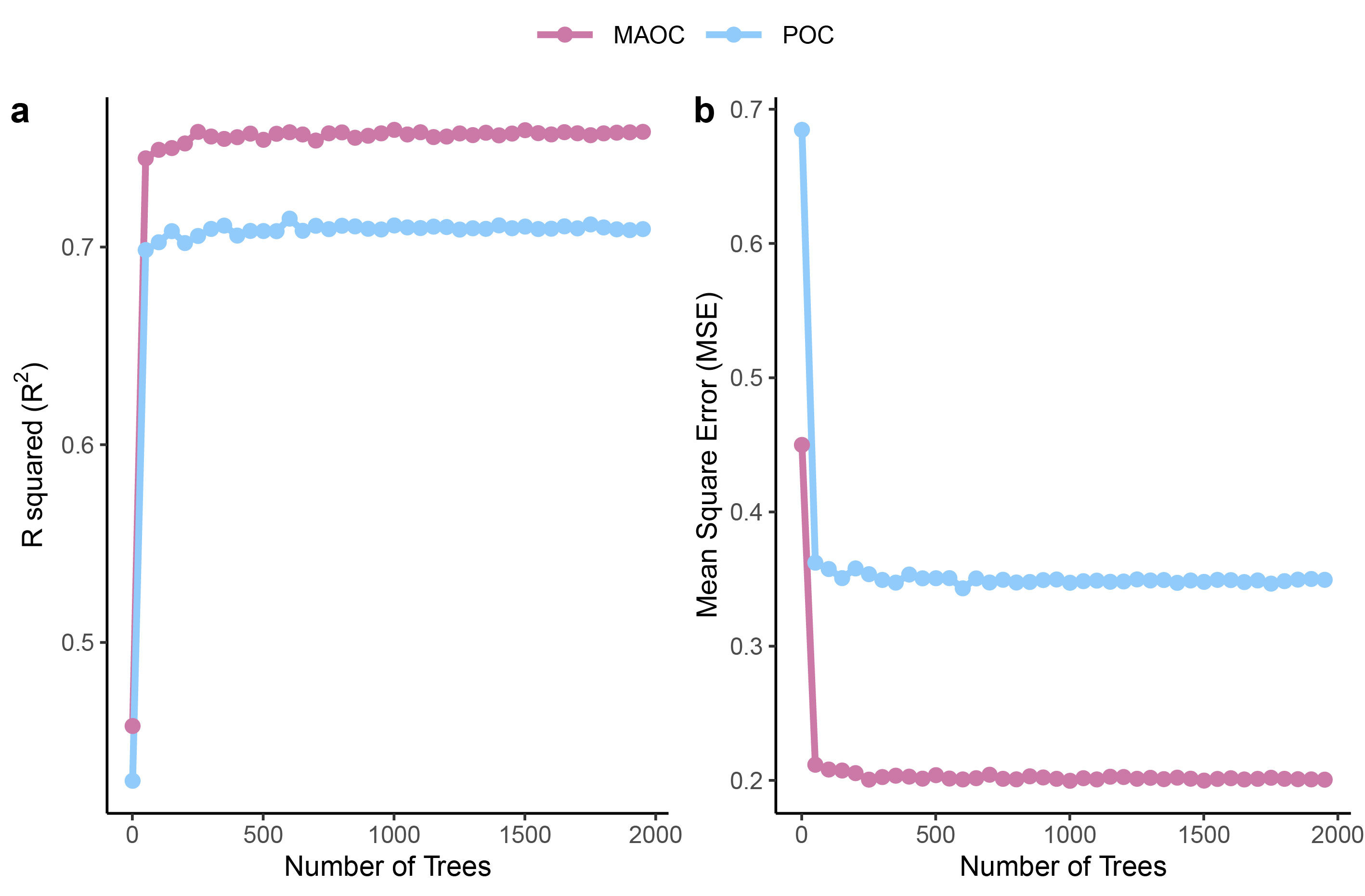
**

**Figure S10.** Spatial autocorrelation analysis of mineral-associated organic carbon (MAOC) and particulate organic carbon (POC). a, c) Semivariograms showing the spatial semivariance of MAOC and POC, respectively, as a function of distance. b, d) Moran’s I values of the random forest model residuals for MAOC and POC, respectively, across distance classes. All Moran’s I values were close to or below zero, and no significant positive spatial autocorrelation was detected, indicating that model residuals were spatially independent.

**
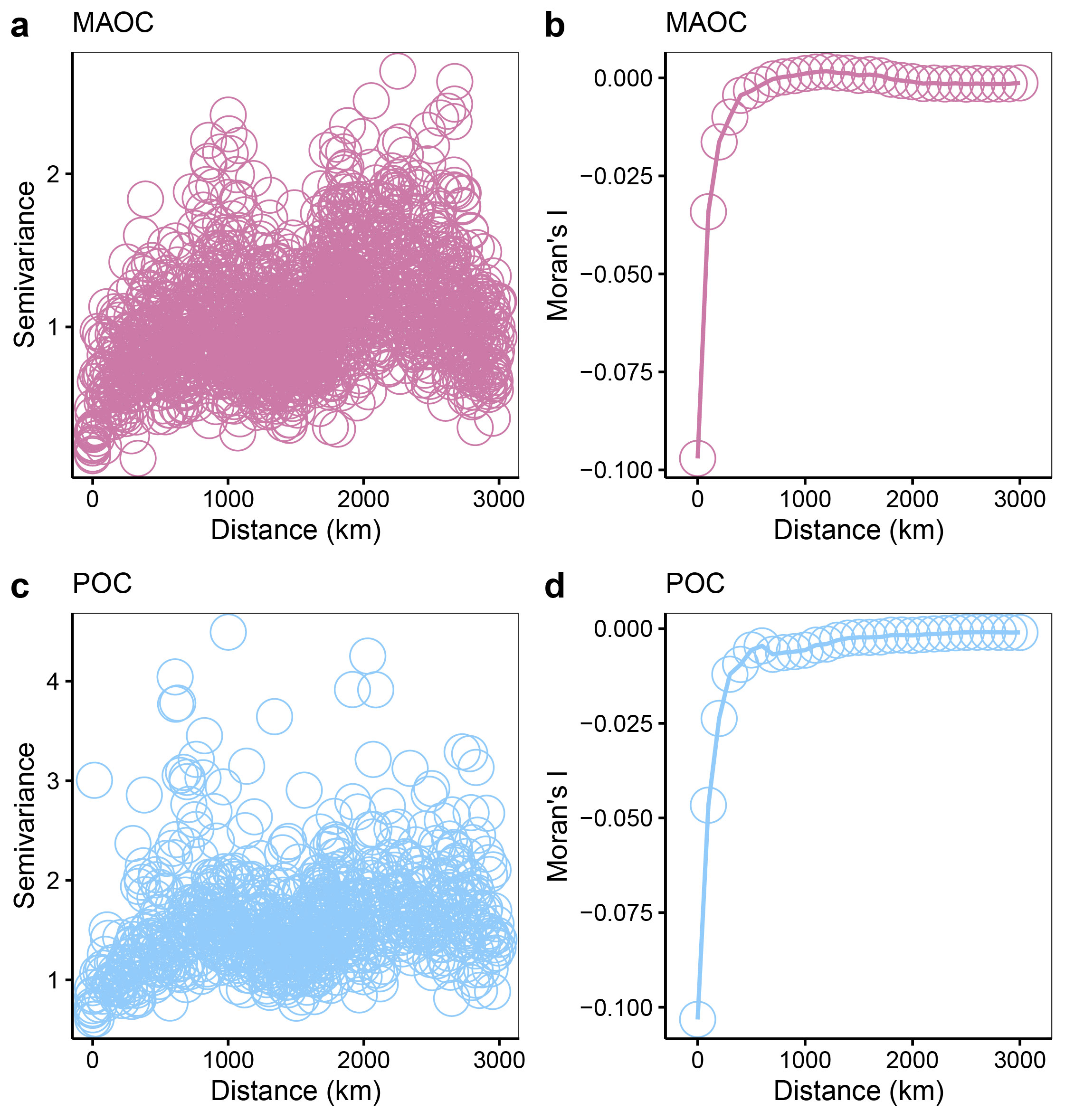
**

**Figure S11.** The workflow predicting soil organic carbon storage potential for China's forests. MAOC, mineral-associated organic carbon; POC, particulate organic carbon.

**
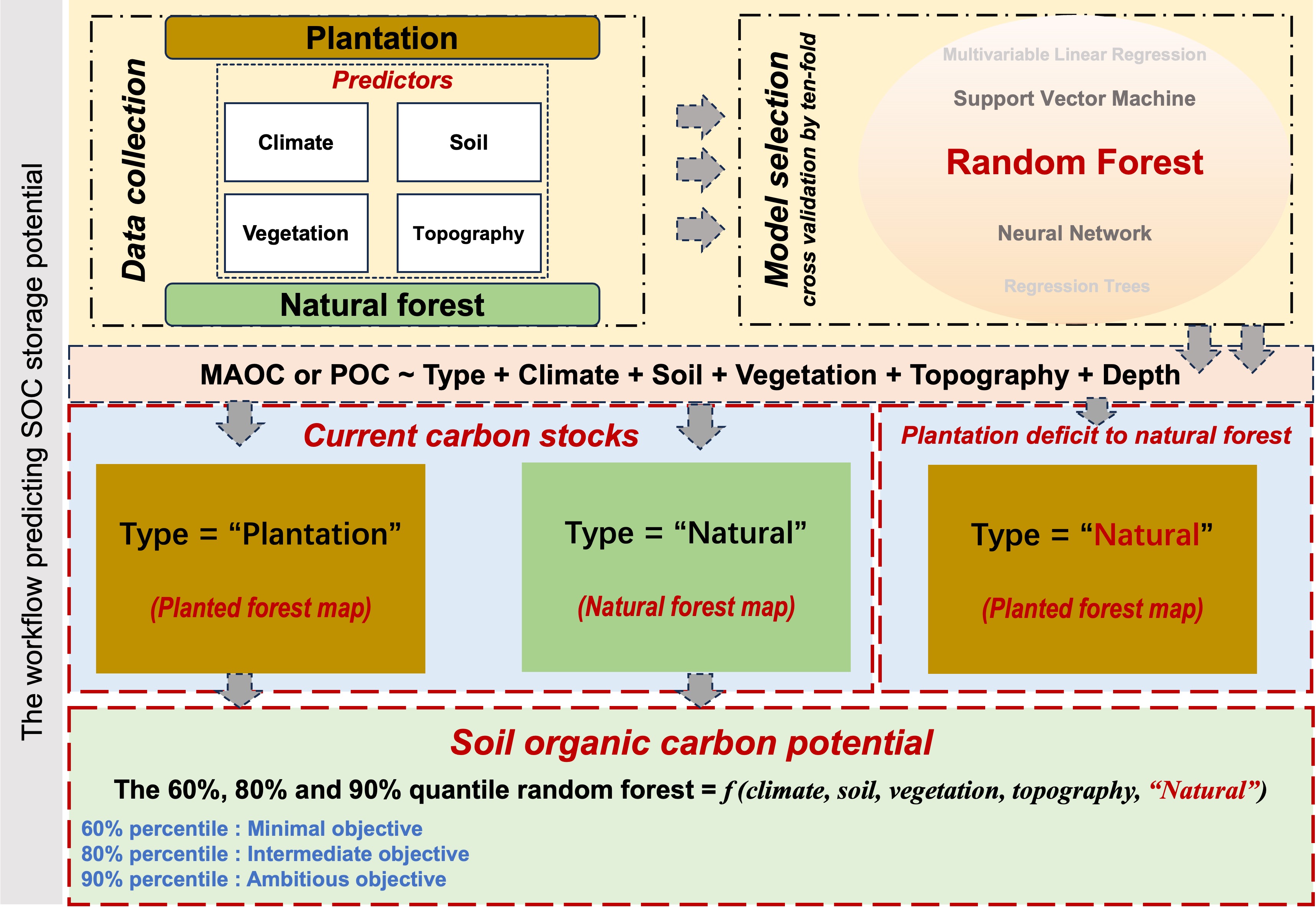
**

**Table S1.** The performance of five machine learning algorithms. *RMSE*, root mean square error. MAOC, mineral-associated organic carbon. POC, particulate organic carbon.

| **Machine learning algorithms** | ***RMSE*** | |
| --- | --- | --- |
|  | MAOC | POC |
| Random forest | 0.192 | 0.310 |
| Regression trees | 0.395 | 0.592 |
| Support vector machine | 0.345 | 0.549 |
| Multivariable linear regression | 0.462 | 0.707 |
| Neural network | 0.805 | 1.040 |
